# Integrative comparison of the genomic and transcriptomic landscape between prostate cancer patients of predominantly African or European genetic ancestry

**DOI:** 10.1371/journal.pgen.1008641

**Published:** 2020-02-14

**Authors:** Jiao Yuan, Kevin H. Kensler, Zhongyi Hu, Youyou Zhang, Tianli Zhang, Junjie Jiang, Mu Xu, Yutian Pan, Meixiao Long, Kathleen T. Montone, Janos L. Tanyi, Yi Fan, Rugang Zhang, Xiaowen Hu, Timothy R. Rebbeck, Lin Zhang

**Affiliations:** 1 Center for Research on Reproduction & Women’s Health, University of Pennsylvania, Philadelphia, Pennsylvania, United States of America; 2 Department of Obstetrics and Gynecology, University of Pennsylvania, Philadelphia, Pennsylvania, United States of America; 3 Department of Medical Oncology, Dana-Farber Cancer Institute, Boston, Massachusetts, United States of America; 4 Department of Epidemiology, Harvard TH Chan School of Public Health, Boston, Massachusetts, United States of America; 5 Department of Internal Medicine, Division of Hematology, Ohio State University, Columbus, Ohio, United States of America; 6 Department of Pathology and Laboratory Medicine, University of Pennsylvania, Philadelphia, Pennsylvania, United States of America; 7 Department of Radiation Oncology, University of Pennsylvania, Philadelphia, Pennsylvania, United States of America; 8 Wistar Institute, Philadelphia, Pennsylvania, United States of America; St Jude Children's Research Hospital, UNITED STATES

## Abstract

Men of predominantly African Ancestry (AA) have higher prostate cancer (CaP) incidence and worse survival than men of predominantly European Ancestry (EA). While socioeconomic factors drive this disparity, genomic factors may also contribute to differences in the incidence and mortality rates. To compare the prevalence of prostate tumor genomic alterations and transcriptomic profiles by patient genetic ancestry, we evaluated genomic profiles from The Cancer Genome Atlas (TCGA) CaP cohort (n = 498). Patient global and local genetic ancestry were estimated by computational algorithms using genotyping data; 414 (83.1%) were EA, 61 (12.2%) were AA, 11 (2.2%) were East Asian Ancestry (EAA), 10 (2.0%) were Native American (NA), and 2 (0.4%) were other ancestry. Genetic ancestry was highly concordant with self-identified race/ethnicity. Subsequent analyses were limited to 61 AA and 414 EA cases. Significant differences were observed by ancestry in the frequency of *SPOP* mutations (20.3% AA vs. 10.0% EA; p = 5.6×10^−03^), *TMPRSS2-ERG* fusions (29.3% AA vs. 39.6% EA; p = 4.4×10^−02^), and *PTEN* deletions/losses (11.5% AA vs. 30.2% EA; p = 3.5×10^−03^). Differentially expressed genes (DEGs) between AAs and EAs showed significant enrichment for prostate eQTL target genes (p = 8.09×10^−48^). Enrichment of highly expressed DEGs for immune-related pathways was observed in AAs, and for PTEN/PI3K signaling in EAs. Nearly one-third of DEGs (31.3%) were long non-coding RNAs (DE-lncRNAs). The proportion of DE-lncRNAs with higher expression in AAs greatly exceeded that with lower expression in AAs (p = 1.2×10^−125^). Both ChIP-seq and RNA-seq data suggested a stronger regulatory role for AR signaling pathways in DE-lncRNAs vs. non-DE-lncRNAs. CaP-related oncogenic lncRNAs, such as *PVT1*, *PCAT1* and *PCAT10/CTBP1-AS*, were found to be more highly expressed in AAs. We report substantial heterogeneity in the prostate tumor genome and transcriptome between EA and AA. These differences may be biological contributors to racial disparities in CaP incidence and outcomes.

## Author summary

Disparities in cancer defined by self-identified race or ethnicity have been a long-standing and persistent challenge. It is well documented that certain racial and ethnic populations in the US experience higher incidence of specific cancer types, higher incidence of aggressive cancers, and higher mortality. The Cancer Genome Atlas (TCGA) data resource contains multi-omic profiles and clinical annotations of large-scale samples, and therefore serves as an excellent resource for the evaluation of the relationship between genetic ancestry and genomic alterations in cancers. In this study, we performed a cancer type specific analysis of the influence of genetic ancestry on genomic alterations in prostate cancers–a malignancy for which there are some of the largest cancer disparities by race and ethnicity in the US. We found that there is substantial heterogeneity in the genomic alterations and transcriptomic dysregulation occurring in men of African (AA) and European (EA) ancestry in the TCGA prostate cancer cohort. *SPOP* mutations, *TMPRSS2-ERG* fusions, *PTEN* deletions/losses, immune signaling, and expression of non-coding RNAs were identified as potential contributors to prostate cancer racial disparities. Our comprehensive characterization of genetic ancestry and genomic/transcriptomic alterations would provide new insight into the biology of prostate cancer racial disparities in the AA population.

## Introduction

Prostate cancer (CaP) is the second leading cause of cancer death in US males, accounting for an estimated 31,620 deaths in 2019 [1]. Incidence, morbidity, and mortality rates of CaP vary substantially by race/ethnicity, representing one of the largest cancer disparities by race/ethnicity in the US [2, 3]. Elevated CaP rates in men of African descent have also been observed globally [4]. While socioeconomic factors and healthcare utilization are significant drivers of these disparities, differences in tumor genomics by race/ethnicity may also contribute to CaP disparities [5]. Mounting evidence suggests that CaPs are phenotypically and genomically heterogeneous by race/ethnicity, potentially signifying differences in tumor etiology [5]. Although genome-wide association studies (GWAS) have identified approximately 200 loci associated with CaP risk and outcomes [6–10], most have not replicated in populations of African descent. GWAS conducted in African men have revealed novel susceptibility loci [11–13]. Recent progress in characterizing CaP genomes [14–19] has motivated efforts to identify genomic contributors to CaP racial disparities. Genomic profiling studies have revealed differences in mutations, copy number alterations, fusions, gene expression, and splicing variants by patient race/ethnicity [20–42]. However, few studies have comprehensively examined CaP heterogeneity by race/ethnicity across multiple genomic platforms within a single sample cohort.

The Cancer Genome Atlas (TCGA) contains data on multidimensional genomic profiles including gene expression, copy number alterations, mutations, transcript fusions, and epigenetic aberrations for 33 common adult cancers, including CaP [14]. Given that a substantial proportion of the US population is represented by genetically admixed populations, these data are a unique resource for the study of cancer racial disparities [43–46]. Recently, we created The Cancer Genetic Ancestry Atlas (TCGAA; http://52.25.87.215/TCGAA/) [47], which provides estimates of genetic ancestry and quantitative ancestral composition for TCGA patients (n = 11,122, involving 33 cancer types from 27 primary sites). We demonstrated that self-identified race/ethnicity (SIRE) and genetic ancestry inferred by our approach were highly correlated across patients. Our estimation of the global and local genetic ancestry for each patient in TCGA, including those patients for whom SIRE is not available, allows for rigorous analysis of the influence of genetic ancestry on genomic alterations across multiple cancer types. For example, we reported that AA patients had higher frequencies of *TP53* mutations and lower frequencies of genomic alterations affecting genes in the PI3K pathway than EA patients [47]. To further interrogate the genomic mechanisms that may contribute to racial disparities in CaP, we compared the frequency of genomic alterations and transcriptomic profiles across groups defined by genomically-inferred ancestry in TCGA CaP cohort.

## Results

### Genetic ancestry and clinical characteristics of the TCGA prostate cancer cohort

**Fig 1:**
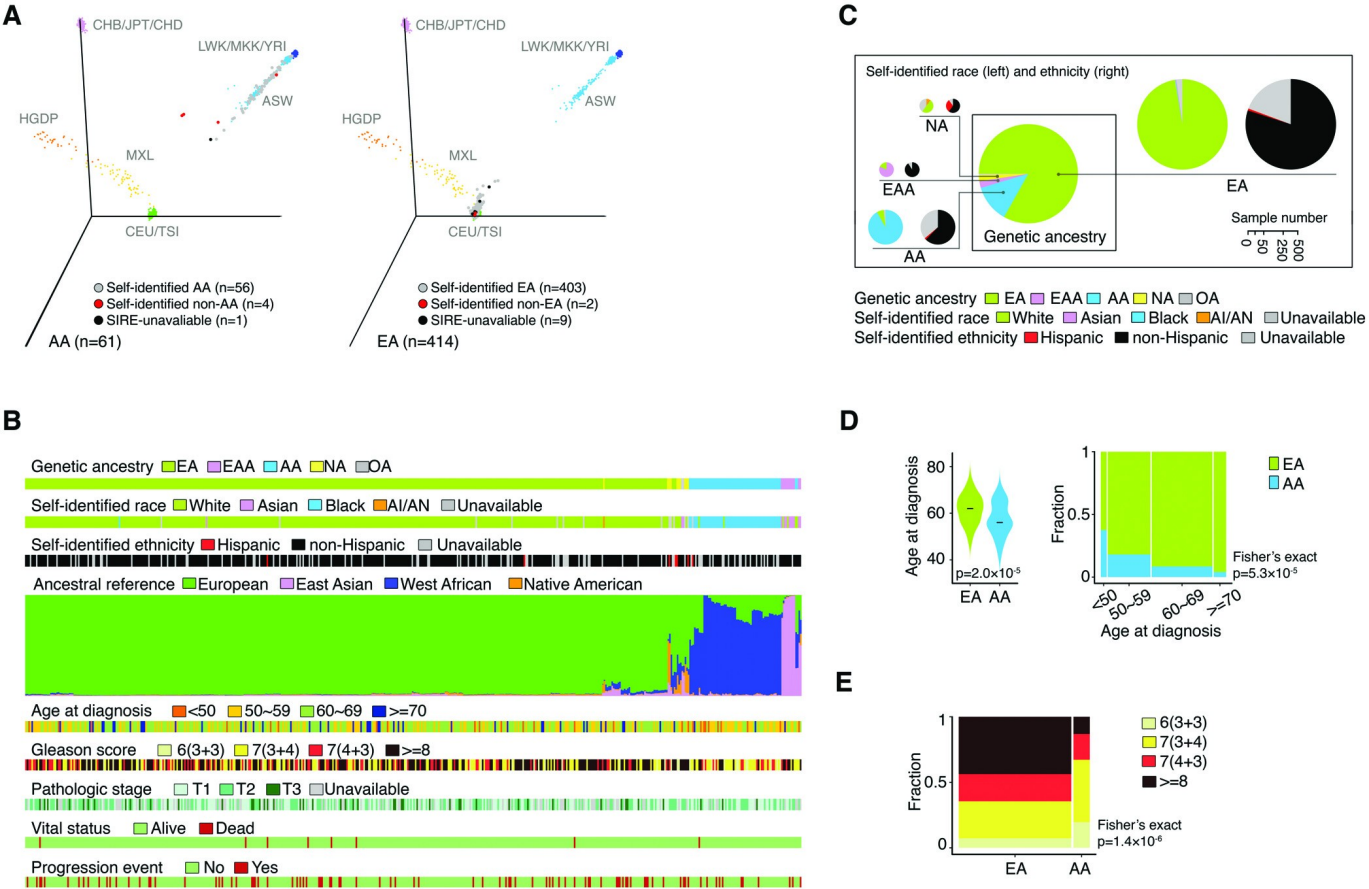
Genetic ancestry and clinical characteristics of the TCGA CaP population. (A) Three-dimensional visualization of genetic variation of EA (left) and AA (right) individuals from the TCGA CaP cohort and the reference populations from the HapMap and HGDP on the first three principle components calculated by EIGENSTRAT. Dot colors show concordance between inferred genetic ancestry and SIRE. (B) Genetic ancestry, SIRE, ancestral composition, and clinical characteristics of the patients in the TCGA CaP sample. Ancestral composition is inferred by STRUCTURE and each color represents an ancestral reference group. Individuals are ordered by hierarchical clustering using Ward's methods on distance matrix calculated as cosine dissimilarity of genetic composition. (C) Pie charts showing agreement between inferred genetic ancestry by EIGENSTRAT and SIRE. Pie chart size is proportional to the number of individuals. (D) Distributions of age at diagnosis among men with AA and EA ancestry. (E) Distribution of Gleason score among men with AA and EA ancestry.

Among the 498 patients with genotyping information (SNP Array) in the TCGA CaP cohort, 83.1% self-identified as White, 11.4% Black, 2.4% Asian, 0.2% American Indian/Alaska Natives. Race was unavailable for 2.8% of patients (S1 Table). In the total cohort, only 1.4% of men self-identified as Hispanic or Latino; self-identified Hispanic or Latino status was unavailable for 20.9% of men. We estimated genetic ancestry for all 498 patients by integrating multiple computational algorithms as described previously [47]. Using EIGENSTRAT and the k-NN algorithm [48], 83.1% of men were classified as EA, 12.2% AA, 2.2% East Asian Ancestry (EAA), 2.0% Native American (NA), and 0.4% other ancestry (S2 Table). Self-identified race/ethnicity (SIRE) and inferred genetic ancestry were concordant for 96.9% of men but differed for four AAs and two EAs (Fig 1A). Next, STRUCTURE [49] and LAMP [50] were used to quantitatively determine the ancestral composition for each patient across the global genome and at particular chromosomal loci (S3 and S4 Tables). As expected, many patients exhibited diversity in ancestral composition inferred by STRUCTURE (Fig 1B), though these estimates were strongly correlated with classifications determined by EIGENSTRAT and SIRE. By integrating the above analyses, we generated a comprehensive view of the distribution of genetic ancestry and SIRE for the TCGA CaP cohort (Fig 1C). Given the limited sample sizes of EAA and NA patients, our subsequent analyses focused on the comparison between AAs and EAs (S1 Fig). We also evaluated the associations between genetic ancestry and clinical characteristics (S5 Table). Briefly, in TCGA prostate cohort, AAs had a lower median age at diagnosis than EAs (56 vs. 62 years, p = 2.0×10^−05^) (Fig 1D), and 13.1% of AAs had tumors with Gleason score 8 or above versus 43.7% of EAs (p = 1.4×10^−06^) (Fig 1E). The distributions of pathological stage, vital status, and progression event status did not differ between AAs and EAs in the TCGA CaP sample. Finally, no significant differences in survival were observed between the two genetic ancestry groups in the TCGA CaP cohort (S1 Fig).

### Genetic ancestry and genomic alterations

**Fig 2:**
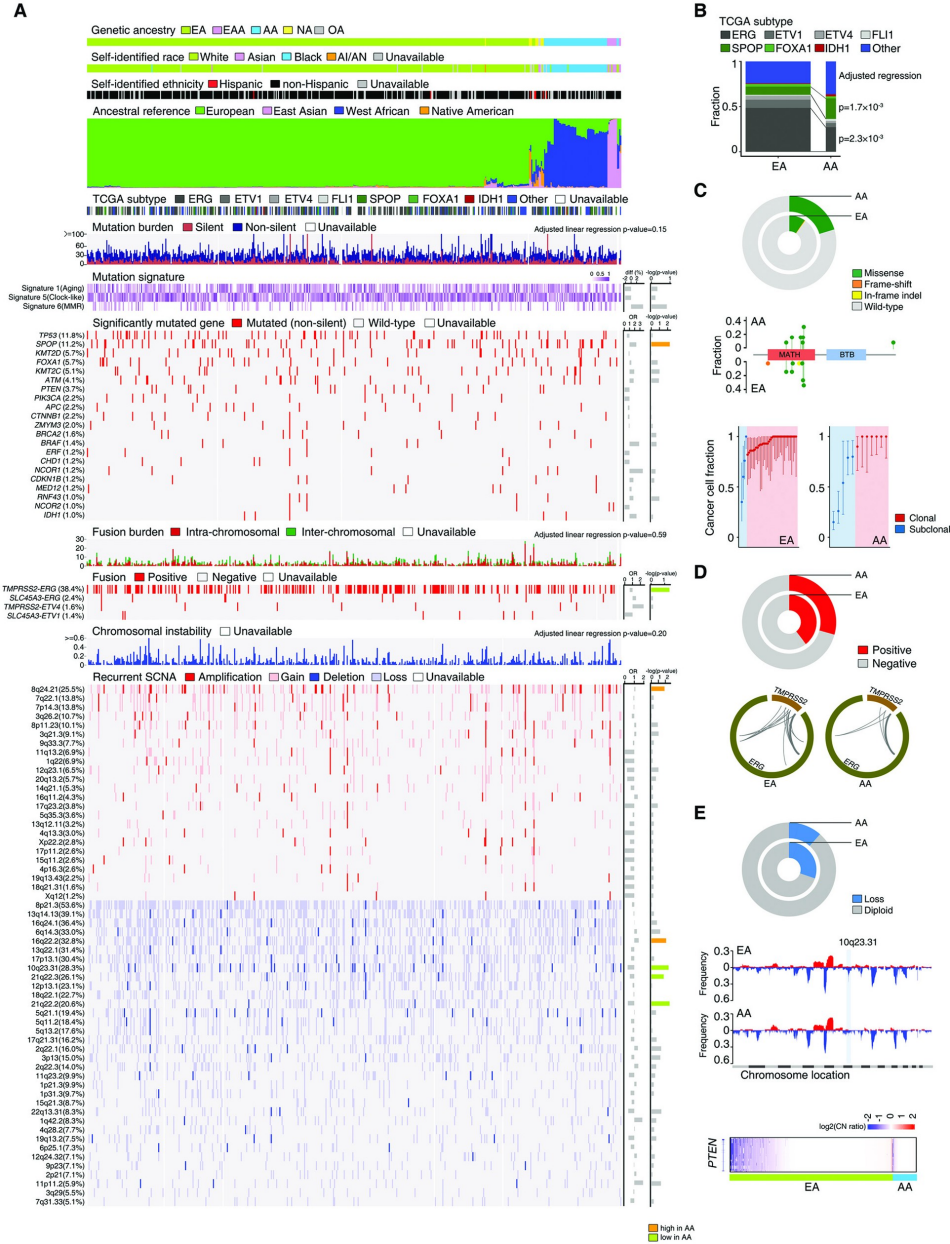
Comparison of genomic alterations by genetic ancestry. (A) Overview of genomic alterations by genetic ancestry in the TCGA CaP cohort. The upper panels show patient genetic ancestry estimated by EIGENSTRAT, SIRE, and ancestral composition estimated by STRUCTURE. Middle panels show participant tumor molecular subtype, mutation burden, presence of mutation signatures 1, 5, and 6, and a heatmap of the mutation frequency at 21 specific genes with ≥1% mutation frequency in the sample. Lower panels show total fusion burden, a heatmap of the frequency of four gene fusions with ≥1% frequency in the sample, total chromosomal instability, and a heatmap of the frequency of 58 recurrent focal somatic copy number alterations (red: amplification/gain; blue: deletion/loss) in the sample. Odds ratios (OR) and differences (diff) compare genomic alteration frequency among men with AA ancestry relative to men with EA ancestry.–log(p-values) are color-coded as significantly more frequent in AA men (orange), significantly less frequent in AA men (green), or no significant difference between AA and EA men (gray). (B) Distributions of seven TCGA CaP molecular subtypes among men with AA and EA ancestry. Men with AA were more likely to have SPOP subtype tumors and less likely to have ERG subtype tumors. (C) Frequency (upper), location (middle), and clonality (lower) of *SPOP* mutations among men with AA and EA ancestry. *SPOP* mutations more frequently occurred in men with AA ancestry (upper). Mutations were preferentially located in the MATH domain for men with AA and EA ancestry. Mutations are color-coded by type: missense (green), frame-shift (red), in-frame insertion/deletion (yellow). Men with AA ancestry had a greater frequency of subclonal *SPOP* mutations than men with EA ancestry (lower). Red represents clonal mutations and blue represents subclonal mutations. (D) Frequency of the *TMPRSS2-ERG* fusion (upper) and distribution of gene breakpoint sites (lower) by genetic ancestry. The *TMPRSS2-ERG* fusion was more prevalent among men with EA ancestry, although breakpoint site patterns were similar among men with EA and AA ancestry. (E) Copy number deletions and losses at 10q23.31 were more common among men with EA ancestry than with AA ancestry (upper). The lower prevalence of 10q23.31 loss for AA men is evident when viewing copy number alterations across the genome (middle). Greater focus on the *PTEN* locus indicates that *PTEN* loss is less common among men with AA ancestry (lower).

The distribution of the seven CaP molecular subtypes defined by the TCGA consortium [14] was compared between AAs and EAs (Fig 2A and 2B). The prevalence of the ERG subtype was 27.3% among AAs and 48.6% among EAs (p = 2.3×10^−03^), while the SPOP subtype was more prevalent among AAs (22.7% vs. 9.1% EA, p = 1.7×10^−03^) (Fig 2B). Next, the spectrum of genomic alterations, including somatic mutations, transcript fusion events, and somatic copy number alterations (SCNAs), was compared between AAs and EAs at both global and individual gene levels (Fig 2A). We fit published mutational signature profiles [51] to somatic single nucleotide variations using the deconstructSigs R package [52]. Mutational signatures 1 (aging), 5 (clock-like) and 6 (mismatch repair [MMR]) were considered (S6 Table), but the contributions of these signatures did not differ between AAs and EAs (all p>0.05). The median mutation burden did not differ between EA and AA patients (p = 0.15). Among 21 CaP genes curated from the published literature [14, 15, 34, 53] with mutation frequency ≥1% in the TCGA CaP cohort, only the frequency of *SPOP* mutations differed significantly between EAs and AAs (p = 5.6×10^−03^) (S7 Table). *SPOP* mutations were present in tumors of 20.3% of AAs and only 10.0% of EAs (Fig 2C). *SPOP* mutations were preferentially found in the MATH domain for both AAs and EAs. Compared to EAs, AAs also harbored a significantly higher subclonal fraction of *SPOP* mutations (p = 8.5×10^−03^), as estimated by the ABSOLUTE algorithm [54]. The total burden of transcript fusion events did not differ by genetic ancestry (p = 0.59); however, the *TMPRSS2-ERG* fusion was more frequently observed in EAs than AAs (39.6% vs. 29.3%, p = 4.4×10^−02^) (S8 Table and Fig 2D). The distribution of *TMPRSS2-ERG* breakpoint sites was similar between EA and AA men. Comparison of the weighted Genome Instability Index (wGII) [55] suggested that global levels of genomic instability and somatic copy number alterations did not differ by ancestry (p = 0.20). Recurrent focal SCNAs were estimated by the Genomic Identification of Significant Targets in Cancer (GISTIC) algorithm [56]. After controlling for clinical factors and global genomic disruption, the frequency of five recurrent focal SCNAs (one amplification and four deletions) significantly differed between EAs and AAs (FDR corrected p<0.25) (S9 Table). For example, the locus encompassing the gene *PTEN* (10q23.31) was less frequently deleted/lost among AAs (p = 3.5×10^−03^) (Fig 2E). Finally, we compared the frequencies of genomic alterations by genetic ancestry among patients with high Gleason score tumors. The observations in the high Gleason groups were consistent with those in the whole TCGA CaP cohort. Additional differential genomic alterations were identified in this subpopulation, including that the mutation frequencies of *KMT2C* and *FOXA1* were significantly higher in AAs with high grade tumors compared to EAs (S2 Fig).

### Genetic ancestry and the transcriptome

**Fig 3:**
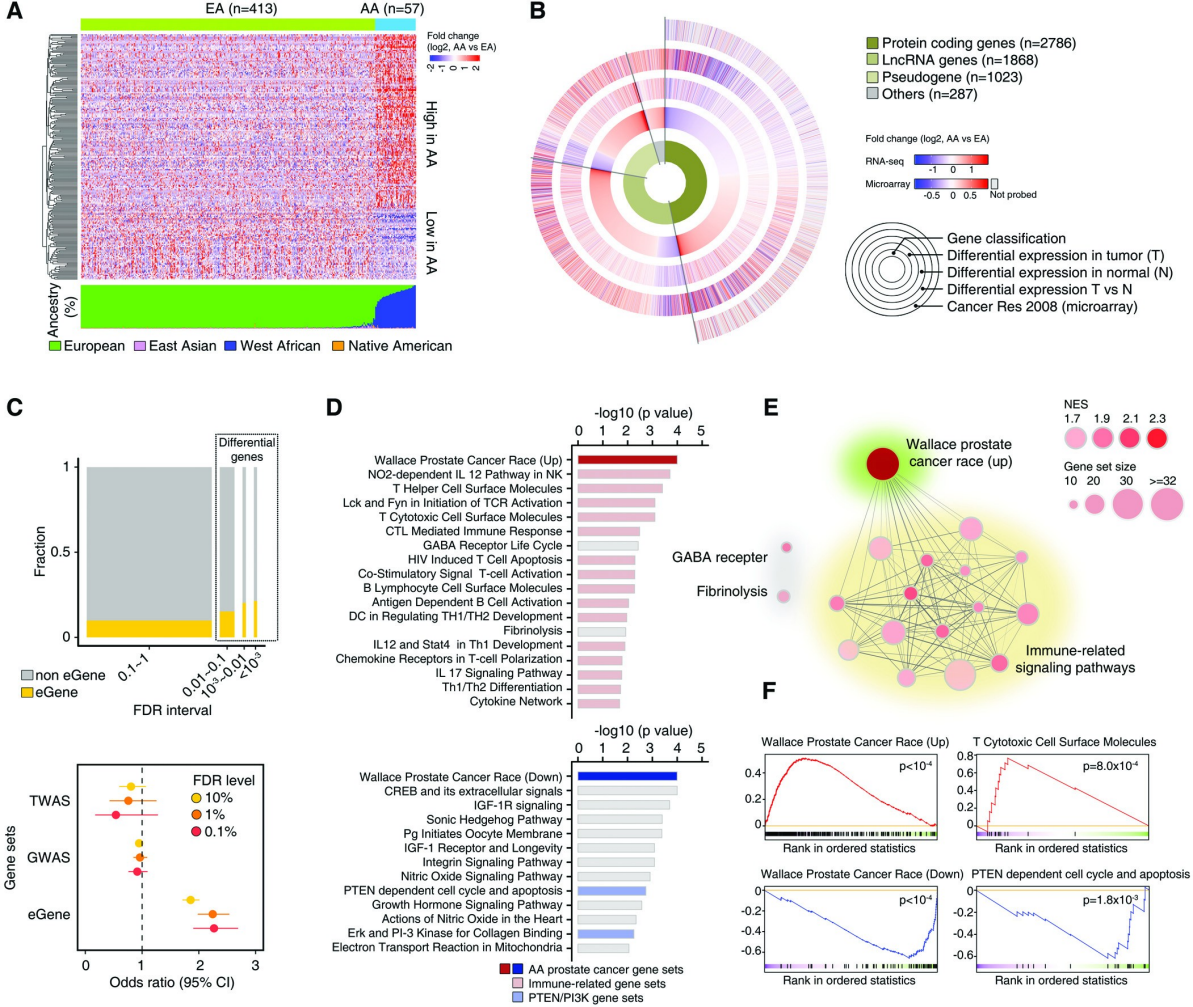
Differences in the prostate tumor transcriptome by genetic ancestry. (A) Heatmap of expression of 220 genes differentially expressed between genetic ancestry groups with fold-change >2. Red denotes a gene more highly expressed in AA men, and blue more highly expressed in EA men. Genetic ancestry is shown using EIGENSTRAT classification (top) and STRUCTURE-estimated composition (bottom). (B) Circle plots showing (from inner to outer): 1) distribution of types of differentially expressed genes (DEGs); 2) fold-change in gene expression comparing AA to EA men in tumor tissue; 3) fold-change in gene expression comparing AA to EA men in tumor-adjacent normal tissue; 4) fold-change in gene expression comparing tumor to tumor-adjacent normal tissue; 5) fold-change in gene expression comparing AA to EA men for protein-coding genes evaluated in Wallace *et al* microarray dataset. (C) Enrichment in DEGs for genes regulated by eQTLs in normal prostate tissues (eGenes) and genes identified in prior GWAS or TWAS of CaP risk. The proportion of eGenes was higher among the DEGs than non-DEGs and increased with more stringent cutoffs in the FDR (upper plot). The prevalence odds of eGenes were higher among the DEGs, while the prevalence odds of GWAS and TWAS genes did not differ between DEGs and non-DEGs (lower plot). (D) Gene sets identified through gene set enrichment analysis that were significantly activated (upper plot; n = 18) and repressed (lower plot; n = 13) in tumors of AA men (FDR corrected p<0.1 through permutation test). Candidate gene sets were identified from the BioCarta pathway database and Wallace *et al*. Many gene sets pertaining to immune-related signaling were activated in AA tumors, while sets related to PTEN/PI3K were repressed in AA tumors. (E) Network plot of the 18 gene sets significantly activated in AA tumors. Nodes are grouped by gene set function. Node size is proportional to the number of genes in the gene set and node shading reflects the Normalized Enrichment Score (NES). An edge indicates there are shared genes between gene sets and edge shading reflects the number of shared genes. (F) Enrichment plots of the Wallace Prostate Cancer Race (Up), T Cytotoxic Cell Surface Molecules, Wallace Prostate Cancer Race (Down), and PTEN dependent cell cycle and apoptosis gene sets.

In the subset of 470 patients (57 AA, 413 EA) with RNA-seq profiling, 3,787 of 60,483 genes (including protein-coding and non-coding genes) were identified as differentially expressed among AAs and EAs using DESeq2 (FDR corrected p<0.1). Upon adjustment for age and Gleason score using a propensity score, 5,964 genes were differentially expressed between AAs and EAs (FDR corrected p<0.1), of which 3,015 were also differentially expressed in the unadjusted analysis (S10 Table). For example, up-regulation of *SPINK1*, a gene defining a molecular subtype of CaP with more rapid progression in an at-risk, natural history radical prostatectomy sample [32, 42], was observed in AA group. Of these 3,015 genes, 220 genes with 2-fold or greater relative expression comparing genetic ancestry groups (69.5% upregulated in AA, 30.5% upregulated in EA) are summarized in the heatmap in Fig 3A. Of the 5,964 differentially expressed genes (DEGs), 46.7% were protein-coding genes and 53.3% were non-coding genes (31.3% lncRNA genes, 17.2% pseudogenes, 4.8% other non-coding) (Fig 3B). In the set of protein-coding DEGs, 50.6% were upregulated in AA and 49.4% were downregulated in AA. In contrast, the majority of non-coding DEGs (82.6%) were upregulated in AA. Of the DEGs, 70.2% (n = 3,984) had race-specific differences in the same direction in the normal tumor-adjacent tissue (OR = 6.2, p = 1.2×10^−205^).The Wallace *et al* [20] microarray dataset was used to validate the findings of differential expression of protein-coding genes by genetic ancestry in TCGA. Of the set of protein-coding DEGs, 61.0% (n = 1,025) had race-specific differences in the same direction in the Wallace dataset (OR = 2.3, p = 4.7×10^−17^). The DEGs identified in TCGA tumor tissue were enriched with genes regulated by normal prostate tissue eQTLs (eGenes, defined by the Genotype-Tissue Expression (GTEx) Project [57]) relative to the non-DEGs (OR = 1.9, p = 8.09×10^−48^) (Fig 3C). Moreover, the DEGs with the most statistically significant differential expression (FDR corrected p<10^−3^) by ancestry showed greater enrichment with eGenes than those with lesser statistical significance. The identified DEGs did not show enrichment for genes whose expression levels were associated with CaP risk through a large-scale TWAS [58] (p = 0.14), or for genes identified in prior GWAS [59] of CaP risk (p = 0.15). Gene set enrichment analysis (GSEA) revealed that 18 and 13 gene sets were significantly activated and repressed, respectively, in AA tumors (FDR corrected p<0.1) (S11 and S12 Tables, Fig 3D). The two gene sets associated with AA and EA race in CaP by Wallace *et al* [20] were most significantly activated and repressed in AAs (both p<10^−4^) (Fig 3D and 3F). Additionally, 15 of the 18 gene sets activated in AAs represented immune-related signaling pathways (Fig 3E). In concordance with the lower prevalence of *PTEN* deletion/loss among AAs in the SCNA analysis, gene sets pertaining to the PTEN/PI3K pathway were repressed in AAs (p = 1.8×10^−3^) (Fig 3D and 3F).

### Genetic ancestry and the non-coding transcriptome

**Fig 4:**
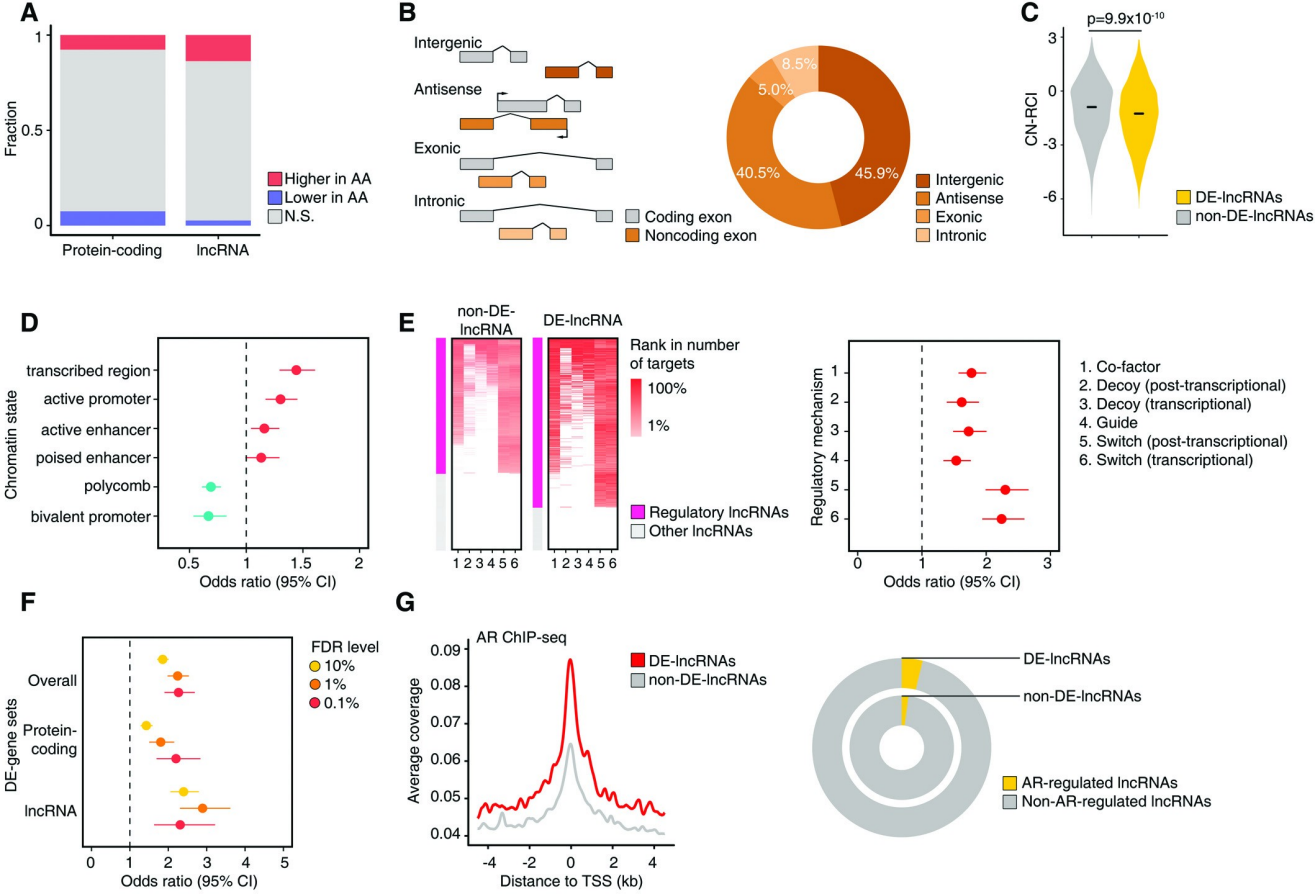
Comparison of the prostate tumor non-coding transcriptome by genetic ancestry. (A) Proportions of DE-protein-coding and DE-non-coding genes. Red indicates statistically significantly higher expression in AA, while blue indicates lower expression in AA. Width of bars is proportional to the number of genes. (B) Classification of lncRNAs by genomic location relative to protein-coding genes (left). Circle plot of distribution of DE-lncRNAs by lncRNA genomic location (right). (C) Distributions of cytoplasmic-nuclear relative concentration index (CN-RCI) for DE-lncRNAs and non-DE-lncRNAs. DE-lncRNAs are more enriched in the nucleus (lower CN-RCI). (D) Odds ratios and 95% confidence intervals (95% CI) of differential expression by genetic ancestry of lncRNAs by chromatin state of the genomic region. Chromatin state is estimated by ChromHMM partitioning of the LNCaP cell. (E) Heatmap displaying enrichment for regulatory lncRNAs among non-DE-lncRNAs and DE-lncRNAs by regulatory mechanism (left). Odds ratios (95% CI) of differential expression by genetic ancestry of lncRNAs by regulatory mechanism (right). (F) Odds ratios (95% CIs) of enrichment with genes regulated by normal prostate tissue eQTLs (eGenes) for DE-genes overall, DE-protein-coding genes, and DE-lncRNAs. Odds ratios are displayed by FDR level of the differentially expressed gene. (G) Average signals of AR binding at transcription start sites (TSSs) of DE-lncRNAs (red) and non-DE-lncRNAs (gray) from AR ChIP-seq data from VCaP cell (left). Circle plot showing proportion of AR-regulated lncRNAs among DE-lncRNAs and non-DE-lncRNAs (right). AR regulation was inferred by analysis of DHT-stimulated transcriptome.

Of the 15,900 manually-annotated and evidence-based human lncRNAs from the GENCODE consortium (version 22), 1,868 (11.7%) lncRNAs were identified as differentially expressed (DE-lncRNAs) between AAs and EAs by DESeq2 (FDR corrected p<0.1) (S13 Table). In contrast to the balance observed between the proportions of coding genes with significantly higher (7.6%) or lower (7.4%) expression in AAs relative to EAs, the proportion of lncRNAs with significantly higher expression in AAs (13.7%) greatly exceeded that with lower expression among AAs (2.6%) (p = 1.2×10^−125^) (Fig 4A). The DE-lncRNAs were classified by their genomic location with respect to protein coding genes and 45.9% were classified as intergenic, suggesting that many of them are transcribed from protein-coding independent transcriptional units and are likely to be functional lncRNAs (Fig 4B). The remaining DE-lncRNAs were classified as 40.5% antisense, 5.0% exonic, and 8.5% intronic. Analysis of the subcellular localization preference of the lncRNAs revealed that DE-lncRNAs had a lower mean cytoplasmic-nuclear concentration index (CN-RCI) than lncRNAs not differentially expressed by genetic ancestry groups (p = 9.9×10^−10^) (Fig 4C). The DE-lncRNAs were more likely to be enriched in the nucleus, supporting their potential function in gene regulation at a transcriptional level. Application of the ChromHMM [60] genomic partitioning of the LNCaP cell line by Valdes-Mora *et al* [61] revealed that the DE-lncRNAs were more likely to be harbored in genomic regions annotated as transcribed, active promoter, and active enhancer compared to the non-DE-lncRNAs (Fig 4D). In contrast, the DE-lncRNAs were less likely to be located around genomic regions annotated as polycomb and bivalent promoter. The DE-lncRNAs were also significantly enriched with regulatory lncRNAs, defined as possessing at least one target gene as predicted by LongHorn [62] (OR = 2.3, p = 2.4×10^−31^) (Fig 4E). The enrichment of regulatory lncRNAs among the DE-lncRNAs was consistent across regulatory mechanisms, although there was greater enrichment for lncRNAs that act as transcriptional and post-transcriptional switches to alter transcription factor or RNA-binding protein activity. Consistent with the results among all DEGs, the DE-lncRNAs were enriched with genes regulated by normal prostate tissue eQTLs (eGenes) relative to non-DE-lncRNAs (p = 4.3×10^−27^), with greater enrichment for eGenes among the lncRNAs than the protein coding genes (lncRNAs OR = 2.4; protein coding genes OR = 1.4) (Fig 4F). However, the DE-lncRNAs did not show enrichment for genes whose expression levels were associated with CaP risk in large-scale TWAS [58] (p = 0.80), or for genes identified in prior GWAS studies [59] of CaP risk (p = 0.47). Using androgen receptor (AR) ChIP-seq data from the VCaP cell line [63], a higher level of AR binding was observed at transcription start sites of DE-lncRNAs relative to the non-DE-lncRNAs (Fig 4G), suggesting greater regulation of the DE-lncRNAs by AR signaling pathways. Among the 1,868 DE-lncRNAs identified by our study, the AR regulatory status for 1,807 of them has been previously characterized by Zhang *et al* [64]. Consistent our findings using the AR ChIP-seq data, 139 (7.7%) were classified by Zhang *et al* as AR-regulated as compared to 4.8% of the non-DE-lncRNAs (OR = 1.6, p = 2.3×10^−6^) (Fig 4G).

**Fig 5:**
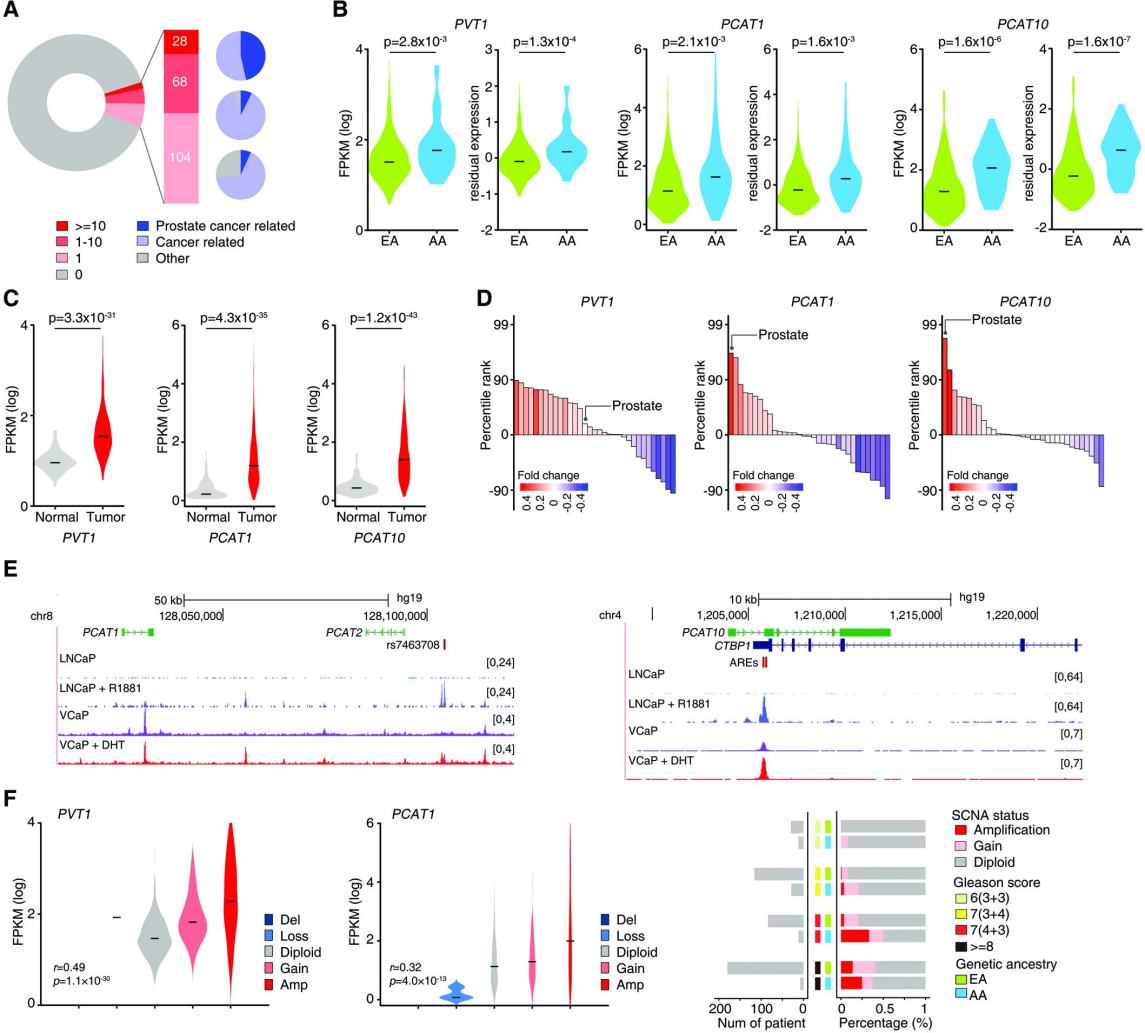
Characterization of the DE-lncRNAs *PVT1*, *PCAT1*, and *PCAT10/CTBP1-AS* in TCGA sample. (A) Circle plot shows the percentage of 1,868 DE-lncRNAs that have been characterized based on related publications in PubMed (left). Red and gray indicate characterized and uncharacterized DE-lncRNAs, respectively. Characterized DE-lncRNAs are defined as the lncRNAs having at least one related publication. The characterized DE-lncRNAs were further ranked into three groups, with a darker shade of red indicating a greater number of related publications. Pie charts show the percentages of the characterized DE-lncRNAs with cancer-related (light blue) or CaP-related (dark blue) results among each group (right). (B) Crude and age- and Gleason score-adjusted expression levels of *PVT1*, *PCAT1*, and *PCAT10* in AA and EA patients. (C) Expression levels of *PVT1*, *PCAT1*, and *PCAT10* in prostate tumors and tumor-adjacent normal prostate tissues. (D) Percentile ranks of enrichment in AA men for *PVT1*, *PCAT1*, and *PCAT10* across 33 TCGA cancer types. Color of bars reflects the fold change of expression (AA vs EA) ranks in a specific cancer type relative to others. High relative expression of *PCAT1* and *PCAT10* among AA men is specific to CaPs, while high relative expression of *PVT1* occurs across cancer types. (E) AR binding signals at genomic loci surrounding or containing *PCAT1* (left) and *PCAT10* (right) from AR ChIP-seq analysis of two CaP cell lines (LNCaP, VCaP) treated with synthetic androgen agonists (R1881, DHT). (F) Expression levels of *PVT1* and *PCAT1* by copy number status (left). Number of men and frequency of SCNAs at 8q24.21 within categories of Gleason score and genetic ancestry (right).

A PubMed search returned one or more related results only for 200 (10.7%) of the 1,868 DE-lncRNAs, suggesting that a clear majority of the DE-lncRNAs are unstudied (Fig 5A), even among the top DE-lncRNAs (S13 Table). Of the 28 DE-lncRNAs that returned ten or more PubMed results, all have been functionally linked to cancer and 13 to CaP specifically. This strongly suggests that these DE-lncRNAs may play important roles in prostate tumorigenesis. For example, the DE-lncRNAs *PVT1*, *PCAT1*, and *PCAT10/CTBP1-AS* have been previously studied and implicated in prostate tumorigenesis [65–70]. These lncRNAs exhibited statistically significantly higher expression among AA patients in crude and age and Gleason-adjusted analyses (Fig 5B; 1.4-fold, 2.1-fold and 1.6-fold, respectively). Moreover, *PVT1*, *PCAT1*, and *PCAT10* were significantly overexpressed in prostate tumors relative to tumor-adjacent normal tissues (Fig 5C). *PCAT1* and *PCAT10* were significantly enriched in a CaP-specific manner relative to enrichment in other cancer types (Fig 5D). In contrast, *PVT1* was highly expressed across multiple cancer types without specificity to CaP. Both *PCAT1* and *PCAT10* have been reported to be AR-regulated [66, 68]. This is further supported by AR ChIP-Seq analysis, which revealed that the AR binding signal was increased in regions containing or surrounding *PCAT1* and *PCAT10* in cell lines treated with synthetic androgen agonists (DHT and R1881) (Fig 5E). Moreover, analysis of the transcriptomic profiles generated by Takayama *et al* [66] further confirmed that expression of *PCAT1* and *PCAT10* was significantly affected by DHT stimulation. Both *PVT1* and *PCAT1* are located within 8q24.21, which is recurrently amplified in CaP, and amplified at a higher frequency among AA patients (p = 9.3×10^−3^) (Fig 5F). There were strong correlations between DNA copy number at 8q24.21 and RNA expression of *PVT1* (*r* = 0.49, p = 1.1×10^−30^) and *PCAT1* (*r* = 0.32, p = 4.0×10^−13^) (Fig 5F). This explains in part the higher expression of *PVT1* and *PCAT1* in AA relative to EA patients.

## Discussion

To further understand how tumor molecular changes may contribute to CaP racial disparities, we comprehensively analyzed the multi-dimensional omics profiles of CaPs by genetic ancestry in TCGA. Our findings buttress existing evidence that patterns of genomic and transcriptomic alterations in prostate tumors differ by patient genetic ancestry [20–42]. For example, transcriptomic analyses revealed strong upregulation of immune-related signaling pathways in AAs relative to EAs. These results are consistent with a growing body of evidence from systems-level gene expression analyses implicating immunobiological dysregulation in CaPs among AAs [20, 21, 23, 30, 38, 39]. The hypothesis that tumor immunobiology differs between AAs and EAs is supported by emerging clinical evidence. In preliminary analyses of the PROCEED Registry, AAs with metastatic castration-resistant CaP receiving the autologous cellular immunotherapy Sipuleucel-T experienced better overall survival relative to EAs [71]. Interestingly, the differentially expressed genes (DEGs) among AA and EA men did not show enrichment for those genes potentially implicated in CaP risk in prior GWAS or TWAS. Although most current GWAS/TWAS were performed in European populations, this finding suggests that the genes potentially driving racial disparities may not be predominately among those identified by GWAS/TWAS. In contrast, we found that DEGs were significantly enriched with eGenes (i.e., genes regulated by normal prostate tissue eQTLs). This suggests that eQTLs and their affected transcripts may largely contribute to CaP racial disparities. Heterogeneity in genetic variants across populations could modulate the transcriptomic background of eGenes, and consequentially these eQTL-regulated DEGs could influence prostate tumorigenesis. Given that prostate eQTLs have been utilized to identify causal genes of CaP risk [72–74], further functional characterization of the DEGs regulated by eQTLs across genetic ancestry groups may provide new insights into molecular mechanism underlying CaP racial disparities.

Our interrogation of the lncRNA transcriptome revealed 1,868 DE-lncRNAs in CaPs between AAs and EAs. Interestingly, nearly 85% of these DE-lncRNAs had higher expression in AAs. Nearly half of the DE-lncRNAs were intergenic, mirroring results from CaP GWAS that identified risk loci in non-coding regions [73, 75]. The localization of these DE-lncRNAs in protein-coding gene deserts supports their expression as independent transcriptional units rather than as artifacts or transcriptional noise from adjacent protein coding genes. Moreover, expression of the DE-lncRNAs was enriched in the nucleus, implicating them in epigenetic transcriptional regulation—one of the major roles of lncRNAs in physiological conditions. Despite their potential functional importance, almost 90% of the identified DE-lncRNAs have not been characterized. Among the 28 highly-studied DE-lncRNAs, all have been previously reported to be involved in cancer, and 13 specifically linked to CaP, strongly suggesting that these DE-lncRNAs may play critical roles in prostate tumorigenesis and potentially contribute to racial disparities. Thus, there is an urgent need for the functional characterization of these understudied DE-lncRNAs, which would assuredly yield novel insights into their functional importance in prostate tumorigenesis and racial disparities. As examples, three functionally characterized and highly expressed in AA DE-lncRNAs, *PVT1*, *PCAT1*, and *PCAT10/CTBP1-AS*, were further studied in the TCGA CaP dataset. *PVT1* and *PCAT1*, located in the vicinity of the *MYC* oncogene, are encompassed by the 8q24.21 protein-coding gene desert–a region harboring many cancer risk variants [76], including CaP risk SNPs in men of African ancestry [77]. We found that this region is more frequently amplified among AA patients, and there are significant and positive correlations between the expression levels of *PVT1*/*PCAT1* and their copy numbers. It suggests that variation in gene copy numbers may partially explain the higher expression of these two DE-lncRNAs in AA patients. Consistently, expression of *PVT1* has been found to be higher in a sample of African Caribbean CaP patients [41]. As a well-known oncogene, *PVT1* promotes tumorigenesis by stabilizing the MYC protein and encoding microRNAs. Additionally, the promoter of the *PVT1* gene regulates *MYC* transcription independently of *PVT1* RNA through competition for shared enhancers [70]. *PCAT1* is regulated by the PRC2 and is highly expressed in a subset of aggressive CaP in which it has a predominantly repressive effect on gene expression [65]. It has been reported that *PCAT1* promotes CaP proliferation and growth by interacting with AR and LSD1 [68]. *PCAT1* is also a potential repressor of *BRCA2*, leading to downstream impairment of DNA damage repair [67]. Finally, the expression levels of *PCAT1* and *PCAT10* may be regulated by AR signaling [66, 68, 78], which was additionally supported by our ChIP-seq analysis. This may explain the CaP specificity of these two DE-lncRNAs, though whether there is differential regulation for their expression by AR among AA and EA patients remains unknown. The totality of evidence from prior work and our own supports multifaceted roles of these three DE-lncRNAs in CaP etiology, suggesting that the further functional characterization of other DE-lncRNAs may provide new insights into the genomic mechanisms of prostate tumorigenesis and the genomic underpinnings of racial disparities.

As we and others have reported before [34, 79], although race and ethnicity were carefully considered during the TCGA sample collection, the absolute sample size of minority populations is still relatively small, generally precluding *de novo* identification of AA population-specific recurrent genomic alterations. The sample size of the TCGA dataset is also insufficient to compare the frequency of uncommon genomic alterations between genetic ancestry groups. For example, although *ERF* was previously identified as recurrently mutated in the tumors of AAs [34], the prevalence of *ERF* mutations did not differ by genetic ancestry in the TCGA cohort. Our analyses adjusted for age and Gleason score, but residual confounding by additional clinicopathological factors is possible. Moreover, information regarding patient socioeconomic status is not available in TCGA, and thus we were unable to account for these important drivers of CaP disparities in our analyses. Racial minority patients are underrepresented in the TCGA CaP cohort and the proportion of AA men with high risk tumors is lower than that among the EA men. Additional genomic data resources designed to address disparities-related questions are still urgently needed to yield a more accurate assessment of the impact of African ancestry, and genetic ancestry generally, on CaP genomic alterations profiles.

In summary, interrogation of the transcriptome in the TCGA CaP dataset revealed that dysregulation of the non-coding transcriptome and genes regulated by prostate eQTLs may contribute to CaP racial disparities. The tumor transcriptome among AAs showed relative enrichment for immune-related pathways. Frequencies of common genomic alterations in CaP including *TMPRSS2-ERG* fusions, *PTEN* deletions/losses, and *SPOP* mutations differed between AAs and EAs. Few of the identified genomic aberrations with differential prevalence across genetic ancestry groups can be therapeutically targeted, and accordingly, future functional characterization is necessary to understand how these factors contribute to prostate tumorigenesis and cancer progression. Moreover, genomic profiling of tumors across racial/ethnic populations is paramount for fulfilling the potential of precision medicine.

## Methods

### TCGA prostate cancer cohort

The genomic profiles and clinical annotations for the TCGA CaP samples were retrieved from the Genomic Data Commons (GDC; https://gdc.cancer.gov/, October 9, 2018). Affymetrix SNP Array 6.0 data in CEL format (n = 498) were downloaded from the TCGA Cancer Genomic Cloud (http://www.cancergenomicscloud.org/). Genotype-calling and subsequent estimation of genetic ancestry was performed as described before [47]. Clinical information was retrieved from GDC (as of October 9, 2018). All TCGA project data used in this study were de-identified. A flow diagram of the study participants used in specific analyses is shown in S3 Fig.

### Associations between genetic ancestry and clinical factors

Differences in the distributions of clinical factors between AAs and EAs were evaluated using the non-parametric Mann-Whitney test for continuous variables (e.g., positive lymph node counts, prostate-specific antigen level, days to biochemical recurrence, and age at diagnosis) or Fisher’s exact test for categorical variables (e.g., Gleason score and pathologic stage). For overall survival and disease-free survival analysis, the log-rank test was used to compare Kaplan-Meier survival curves and the Mantel-Haenszel method was used to estimate hazard ratios comparing AAs and EAs. All statistical tests were two-sided.

### Adjustment for clinical factors

Clinical factors were accounted for in all analyses comparing genetic characteristics between AA and EA patients. Age at diagnosis (mean, 61.0 years; range, 41–78 years) and Gleason score (< = 6, 9.0%; 7(3+4), 29.8%; 7(4+3), 20.2%; > = 8, 41.0%) were comprehensively annotated [14] and their distributions significantly differed between AA and EA patients. These two factors were used to derive a propensity score [80], treating age as a continuous variable and Gleason score as an ordinal categorical variable. The propensity score [80] was then included as a covariate in regression models testing the associations between ancestry and genetic characteristics (e.g., overall SCNA scores [81], alteration status of somatic events and expression signature scores). Propensity scores were estimated separately in the context of each molecular platform.

### Estimation of global and local genetic ancestry

The methods used to estimate global and local genetic ancestry for the TCGA CaP sample have been described previously [47]. Briefly, unrelated individuals from HapMap (n = 1,117) and HGDP (Native American panel, n = 64) were used as reference populations, which were further grouped into 7 populations according to continental distribution and migration history: West Africans (YRI), European (CEU and TSI), East Asian (CHB, CHD and JPT), Native American (Pima, Maya, Colombians, Karitiana and Surui), South Asian (GIH), African American (ASW, LWK and MKK), and Mexican (MEX). The smartpca program of the EIGENSTRAT algorithm [48] was applied to run PCA on the combined genotype data of TCGA patients and the reference populations. Eigenvectors were computed using reference populations only, by specifying a poplist file using the -w option. The program then output the position of each individual (either from TCGA or from reference populations) on the top ten axes of variation into a file with the extension .evec. This allowed for visualization of the population structure as well as estimation of relative distance between individuals or populations. Applying a k-nearest neighbor (k-NN) classifier to find the nearest neighbors for each of the TCGA patients, we categorized TCGA patients into one of five genetic ancestry groups: European American (EA), African American (AA), East Asian American (EAA), Native American (NA), and other ancestry (OA). Priority of sample type was blood > solid normal > tumor. In addition, to investigate genetic variation within populations, STRUCTURE [49, 82, 83] was used to yield a quantitative estimate of each individual’s ancestral composition and LAMP [50] was used to yield an estimation of genetic ancestry at specific chromosomal loci.

### Molecular subtypes

The prevalence of the seven molecular subtypes previously identified in the TCGA CaP sample was compared between men of AA and EA ancestry [14]. The subtypes are defined by the presence of ETS fusions (ERG, ETV1, ETV4 and FLI1) or mutations in SPOP, FOXA1, and IDH1. Subtype frequencies by genetic ancestry were evaluated using Fisher's Exact Test and a logistic regression model adjusted for age and Gleason score.

### Somatic mutation signature analysis

Mutation annotation files (MAFs) were downloaded from the GDC Data Portal (https://portal.gdc.cancer.gov/ using the Genomic Data Commons (GDC) Data Transfer Tool (gdc-client_v1.2.0_Ubuntu14.04_x64). Single nucleotide variants (SNVs) of somatic mutations generated by the MuTect2 pipeline were identified using the MAFs. All SNVs of somatic mutations that passed quality control were included in mutational signature analyses. Each mutation was classified into one of six base substitution categories (C>A, C>G, C>T, T>A, T>C, and T>G) and the sequence context in which a mutation occurred (the bases immediately 5′ and 3′ of the mutated base; 16 possible combinations) was also considered. Thus, the mutational spectrum of a tumor can be summarized as a vector of length 96, where each element represents the mutation count at one of the 96 mutated trinucleotides. A set of consensus mutational signatures identified from *de novo* extraction across 10,250 samples was published by the Wellcome Trust Sanger Institute (WTSI) Mutational Signature Framework [51]. Using the set of signatures, which are defined as prevalent in specific tumor types, deconstructSigs [52] fit a multiple linear regression model with the constraint that any coefficient must be non-negative to most accurately reconstruct the observed mutational spectra. The weights of signatures were normalized between 0 and 1 with the sum of normalized weights equal to 1. By default, in deconstructSigs, any signature contribution with a weight less than 0.06 is discarded. Cosine similarity was used to compare the mutational spectra of the original and reconstructed samples. Signatures that failed to improve cosine similarity by more than 0.02 were removed, and the sample was reanalyzed with the remaining signatures. In the CaP dataset, the age-associated signatures (signatures 1 and 5) were dominant, while the defective DNA mismatch repair (MMR)-associated signature (signature 6) also contributed to the mutational spectrum of a subset of patients. The differences in the contributions of each mutational signature among AA and EA patients were assessed using a model regressing the contribution of each signature on AA ancestry status (AA as 1 while EA as 0) adjusting for age and Gleason score. The Benjamini and Hochberg (BH) procedure was applied to control the false discovery rate (FDR) [84].

### Somatic mutation burden analysis

Mutation burden was defined as the total number of somatic mutations present in a tumor specimen. A linear regression model was fit to evaluate genetic ancestry as a predictor of rank-normalized mutation burden, adjusting for the propensity score.

### Recurrently mutated gene analysis

A list of 45 recurrently-mutated CaP genes was curated from published literature [14, 15, 34, 53]. For analysis of individual CaP genes, the TCGA PanCanAtlas MC3 dataset was downloaded as the mutation call set [85]. Only non-silent mutations were included in downstream analyses. Mutations in individual patient samples were aggregated to gene-level. The mutation frequency of a gene was defined as the proportion of individuals in the cohort harboring mutations. Twenty-one CaP genes had mutation frequency ≥1% in the TCGA CaP cohort and were considered in subsequent analyses. The association between mutation frequency and genetic ancestry was assessed using logistic regression models adjusted for age and Gleason score. The Benjamini and Hochberg (BH) procedure was applied to control the false discovery rate (FDR) [84]. Mutations were classified as clonal or subclonal using the ABSOLUTE algorithm [54]. ABSOLUTE integrates genome-wide copy-number data from SNP arrays and the allelic fraction values of somatic mutations to model gene and mutation copy number in a tumor. The frequencies of clonal/subclonal mutations by genetic ancestry were evaluated using Fisher’s Exact Test.

### Gene fusion analysis

Gene fusion data for TCGA were retrieved from Gao *et al* [86]. Fusion burden was defined as the total number of gene fusion events present in a tumor specimen. Linear regression was used to evaluate the association between genetic ancestry and rank-normalized fusion burden, adjusting for clinical covariates. In addition, the association between genetic ancestry and the prevalence of individual fusion events was tested in logistic regression models where the dependent variable was the presence of a specific fusion event and the age- and Gleason score-derived propensity score was supplied as a covariate. Only fusion events with frequency ≥1% in the TCGA CaP sample were compared. The Benjamini and Hochberg (BH) procedure was applied to control the false discovery rate (FDR) [84].

### Recurrent focal SCNA analysis

Segmentation files for somatic copy number alteration (SCNA) analysis were obtained from the TCGA Cloud (http://www.cancergenomicscloud.org/) for 494 patients (including 61 AA and 410 EA). For each patient, a pair of segmentation files of tumor and matched control (if available) was selected for somatic copy number alteration analysis. If multiple aliquot barcodes existed for one patient, one single pair of tumor/matched control samples was selected with the following hierarchy: (1) sample type: for tumor tissues, primary > recurrent > metastatic (Sample Type code: 01 > 02 > 06); for normal control tissues, blood > solid (Sample Type code: 10 > 11); (2) molecular type of analyte for analysis: prefer D analytes (native DNA) over G, W, or X (whole-genome amplified); (3) order of sample portions: higher portion numbers were selected; and (4) order of plate: higher plate numbers were selected. Recurrent focal SCNAs (peak regions) were identified using GISTIC 2.0 (ftp://ftp.broadinstitute.org/pub/GISTIC2.0/) [56]. After excluding tumors with more than 2,000 segments, significant peak regions were identified with q value < 0.25. The confidence level used to calculate the region containing a driver was set to 0.95 (by the–conf option). Fifty-eight total recurrent focal SCNAs were identified in the TCGA CaP dataset. We carefully examined the location of each peak region and no peaks were located in centromeres or telomeres (within 1 Mb).

The relative frequency of focal SCNA events at each peak region was compared between AA and EA men using logistic regression adjusting for age and Gleason score. To control the overall level of genomic disruption when comparing the alteration frequencies for each SCNA event across ancestry groups, we performed a controlled permutation test in which both the fractions of the genome affected by each of the amplifications and deletions in each sample (column-wise) and the alteration frequency of each recurrent focal SCNA (row-wise) were maintained in the permuted data. We first created a binary matrix *X*∈*R^n×m^* denoting the recurrent focal SCNA profile across all tumors, where *n* is the number of tumors, *m* is the total number of recurrent focal SCNAs and the (*i,j*)^*th*^ element of the matrix *X, X_ij_* is determined following: $X_{ij}=\left\{ \begin{array}{cc} 1 & c_{ij}\geq 0.25\;and\;j^{th}\;focal\;SCNA\;is\;recurrent\;amplified\\ 1 & c_{ij}\leq -0.25\;and\;j^{th}\;focal\;SCNA\;is\;recurrent\;deleted\\ 0 & other \end{array}\right.$, in which *c_ij_* is the actual change in copy number for *j^th^* recurrent focal SCNA in *i^th^* tumor. We created 10,000 permutations of the matrix using the function permatswap() in the R package vegan. The genomic disruption level of each tumor (row-wise sum of the matrix) as well as each recurrent focal SCNA (column-wise sum of the matrix) were maintained in the permutations by supplying the parameters: method = "quasiswap", fixedmar = "both", shuffle = "both" and mtype = "prab". Permutation was performed within amplifications and deletions separately. A test statistic for the association with AA patients relative to EA patients for each recurrent focal SCNA was generated using a logistic regression model for each permutated recurrent focal SCNA profile. This model used AA ancestry status (AA as 1, while EA as 0) as the dependent variable and the binary alteration status as the independent variable, adjusting for the propensity score as a covariate. Subsequently, the significance (Z score and raw p value) of the association between each recurrent focal SCNA and genetic ancestry was determined by examining the position of the observed test statistic among the distribution of all statistics generated from the permutated recurrent focal SCNA profiles. The Benjamini and Hochberg (BH) procedure [84] was then applied to the set of raw p values to control the FDR.

### Weighted genome instability index (wGII) analysis

To assess chromosomal instability, the weighted Genome Instability Index (wGII) was calculated using segmentation files [55]. First, the ploidy of each tumor was assigned as the median copy number accounting for the length of segments. For each chromosome, GII was calculated as the fraction of the genome presenting aberrant copy numbers (differing more than 0.3) relative to baseline ploidy. The wGII of the tumor was calculated as the mean fraction of aberration across all 22 chromosomes, such that all chromosomes influence the score equally regardless of size. The association between genetic ancestry and wGII was evaluated using a linear regression model where the dependent variable was wGII and the independent variable was AA status (AA as 1 and EA as 0), adjusting for the propensity score.

### Gene expression analysis

Gene-level RNA-Seq data were downloaded using the Genomic Data Commons (GDC) Data Transfer Tool (gdc-client_v1.2.0_Ubuntu14.04_x64) from the GDC Data Portal (https://portal.gdc.cancer.gov/). In the GDC RNA-seq analysis pipeline, reads were aligned to the GRCh38 reference genome, and then gene level expression was measured from HT-Seq raw read count using GENCODE v22 for gene annotation. For tumor expression analyses, one single aliquot barcode was selected for each patient with the following selection hierarchy: (1) sample type: primary > recurrent > metastatic (Sample Type code: 01 > 02 > 06); (2) molecular type of analyte: prefer R analytes (RNA) over T (Total RNA); (3) order of sample portions: higher portion numbers were selected; and (4) order of plate: higher plate numbers were selected. Differential gene expression analysis between tumor samples from AA and EA patients was performed using the DESeq2 package [87] with the raw count matrix as input, adjusting for potential confounding by age and Gleason score. Similar procedures were applied for differential gene expression analysis comparing AA and EA patients in tumor-adjacent normal tissues, although analyses adjusted for age only. In addition, genes differentially expressed between tumor and adjacent normal tissues were also identified. As an external validation cohort, the microarray dataset from Wallace *et al* [20] was retrieved from the Gene Expression Omnibus (GEO) database: GSE6956 (Platform: GPL571; Affymetrix Human Genome U133A 2.0 Array; 33 AA vs. 37 EA). Differentially expressed genes were identified using the GEO2R analyzer.

### Functional enrichment for differentially expressed genes (DEGs)

Genes regulated by normal prostate tissue eQTLs (eGenes) were identified by the Genotype-Tissue Expression (GTEx) Project [57]. The list of eGenes (Release V7; qval < = 0.05) for prostate tissue was obtained from the GTEx Portal (https://gtexportal.org/). GWAS-related genes were defined as those located within 1Mb of GWAS SNPs. GWAS SNPs associated with CaP risk were retrieved from the NHGRI-EBI GWAS Catalog [59]. TWAS-related genes, those whose expression levels were associated with CaP risk, were identified from a prior large-scale TWAS study [58]. Enrichment for eGenes, GWAS-related, and TWAS-related genes among DEGs compared to non-DEGs was assessed by Fisher’s Exact test using variable FDR thresholds (0.1, 0.01, 0.001). To identify specific gene sets that were differentially regulated in AA and EA men, we performed gene set enrichment analysis (GSEA) using the BioCarta pathways (http://www.biocarta.com/) and the CaP disparity-related gene sets previously reported by Wallace *et al* [20]. GSEA was performed using the GSEA pre-ranked module on GenePattern (https://genepattern.broadinstitute.org/). A gene list ranked by the Wald statistic calculated by DESeq2 was used. Visualization of significantly enriched gene sets was performed using Cytoscape (v3.5) [88].

### LncRNA classification

In an effort to classify lncRNA genes and reveal their potential biological roles, we determined their genomic distribution patterns relative to protein-coding loci. We first defined two classes of lncRNA genes, intergenic and genic, according to whether their boundaries overlapped with any protein-coding genes. The set of genic lncRNAs was further classified into three locus biotypes. First, we identified lncRNAs transcribed in an antisense direction and designated them as the “antisense” biotype. For lncRNAs transcribed in the same direction as the nearest gene, they could overlap protein-coding genes, sharing exonic regions (the “exonic” biotype) or intronic regions (the “intronic” biotype). LncRNAs were evaluated at a transcript level. In cases where a lncRNA gene had multiple transcripts that could be classified into different types, the hierarchy of types used to classify lncRNAs was: exonic > intronic > antisense > intergenic.

### LncRNA regulatory potential

The subcellular localization of lncRNAs can provide insight into their molecular function and we therefore compared the relative concentration index (RCI) between lncRNAs differentially expressed and non-differentially expressed with respect to genetic ancestry. RCI values were downloaded from the lncATLAS database [89]. A lower RCI value indicates enriched localization in the nucleus. RCI values of the differentially-expressed and non-differentially expressed lncRNAs were compared by Student’s *t*-test. Annotation of the genomic position also facilitates the functional interpretation of lncRNAs. Applying ChromHMM [60], Valdes-Mora *et al* [61] partitioned the LNCaP cell line into seven distinct states: active promoter, bivalent promoter, active enhancer, poised enhancer, polycomb, transcribed region, and unmarked region. A lncRNA was defined as related to a specific epigenetic state if it was located within 10kb of any of the regions annotated as that state. Enrichment for each epigenetic state among differentially-expressed and non-differentially expressed lncRNAs was evaluated using Fisher’s Exact test. Additionally, Chiu *et al* [62] previously suggested potential regulatory functions of lncRNAs in context of prostate tumors. Enrichment for overall regulatory lncRNAs and those within different mechanisms, as classified by Chiu *et al*, among differentially expressed and non-differentially expressed lncRNAs was assessed by Fisher’s Exact test. The Benjamini and Hochberg (BH) procedure [84] was applied to control the FDR.

### Androgen receptor (AR)-regulation potential of differentially-expressed lncRNAs

AR ChIP–seq data performed on the VCaP and LNCaP CaP cell lines from published datasets were downloaded from the GEO (GSE55064) and Nuclear Receptor Cistrome (NRCistrome, http://cistrome.dfci.harvard.edu/NR_Cistrome/). Wig profiles (assembly GRCh37/hg19) were also downloaded. The AR binding signal across lncRNAs of interest was visualized using the UCSC genome browser. The aggregation plot of ChIP-seq signals across transcriptional start sites (TSS) of differentially-expressed and non-differentially expressed lncRNAs was generated by agplus [90]. RNA-seq profiling data of AR-dependent VCaP and LNCaP CaP cell lines that were stimulated with an AR ligand, dihydrotestosterone (DHT), were obtained from GEO (GSE110905). Eighteen genes that are experimentally-validated AR transcriptional targets [14, 53] were used as positive control to find a reasonable cutoff to classify AR-regulated genes (ARGs) based on transcriptome analysis. Applying a cutoff of a 1.2-fold change, 16 (88.9%) experimentally-validated AR transcriptional target genes had elevated expression levels after DHT stimulation. Thus 1.2-fold (either enhanced or suppressed) was used to classify genes as AR-regulated. Enrichment of ARGs among differentially-expressed and non-differentially expressed lncRNAs was compared using Fisher’s Exact test.

### Literature search

In order to investigate the extent to which the DE-lncRNAs had been previously studied, a custom script was executed to search each lncRNA in PubMed and record the number of articles returned. The name of each DE-lncRNA was first searched alone, and then in combination with "cancer" and "prostate cancer".

### Lineage specificity of lncRNAs

In order to characterize which CaP disparity genes were specific to CaP or common across multiple cancer types, we assayed lineage specificity using sample set enrichment analysis (SSEA), a method adapted from Gene Set Enrichment Analysis (GSEA). Tumors within TCGA were ordered according to the expression level of a specific lncRNA while a sample set was defined by a specific cancer type. Enrichment scores (ES) and normalized enrichment scores (NES) were calculated in the same way as in GSEA. Thirty-three SSEA analyses were performed for every lncRNA comparing each cancer type to the others within TCGA. Finally, we ranked genes within each cancer type by normalized enrichment score (NES) and assigned fractional ranks (for example, a fractional rank of 0.95 implies that the gene ranked in the top 5th percentile of all genes in the cancer type) to assess cancer type specificity.

## Supporting information

S1 Fig Survival analysis within The Cancer Genome Atlas (TCGA) prostate cancer dataset. (A) Censoring rates of OS, DSS, DFI, and PFI. (B) Forest plot showing the association between African ancestry and prostate cancer survival outcomes. Dots and horizontal lines represent HR values and their 95% CIs, respectively. Arrows indicate where the CI values extend outside the range indicated. P-values were calculated from the Wald test of the coefficient for genetic ancestry in the Cox PH models. (C-F) Genomic alterations associated with (C) OS, (D) DSS, (E) DFI, and (F) PFI. The bar plots show the significance (y axis) for each genetic event of somatic mutations, transcript fusions, recurrent focal amplifications, and recurrent focal deletions. Orange and green bars represent the genetic events that had significantly higher or lower alteration frequencies among patients with worse survival outcomes, respectively. Genomic alterations with non-significant results are colored in gray.
(TIF)

S2 Fig Comparison of genomic alterations and the transcriptome by genetic ancestry within high risk patients (Gleason score 4+3 or higher). (A) Comparison of genomic alterations by genetic ancestry. The bar plots show the significance (y axis) for each genetic event of somatic mutations, transcript fusions, recurrent focal amplifications, and recurrent focal deletions. Orange and green bars represent the genetic events with alteration frequencies that were significantly higher or lower in patients of AA ancestry, respectively. Genomic alterations with non-significant results are colored in gray. (B) Mutation frequency of *SPOP*, *KMT2C*, and *FOXA1* stratified by genetic ancestry. (C) Frequency of *TMPRSS2*-*ERG* fusion stratified by genetic ancestry in high risk prostate cancer patients. (D) Frequency of 2q22.1 (*SPOPL*), 10q23.31 (*PTEN*), 6q14.3 (*ZNF292*) and 21q22.3 (*TMPRSS2*) copy number alterations stratified by genetic ancestry. (E) Gene sets identified through gene set enrichment analysis that were significantly activated (upper plot; n = 13) and repressed (lower plot; n = 2) in tumors of AA men (FDR corrected p<0.1 through permutation test). Candidate gene sets were identified from the BioCarta pathway database and Wallace et al. Many gene sets pertaining to immune-related signaling were activated in AA tumors. (F) Enrichment plots of the Wallace Prostate Cancer Race (Up), T Cytotoxic Cell Surface Molecules, Wallace Prostate Cancer Race (Down), and CARM1 and Regulation of the Estrogen Receptor gene sets.
(TIF)

S3 Fig Flow diagram for identification of analytic population within The Cancer Genome Atlas (TCGA) prostate cancer dataset. Clinical and genomic data were retrieved for 498 patients. EIGENSTRAT and a k-nearest neighbors (k-NN) classifier were applied to classify individuals as: European American (EA), African American (AA), East Asian American (EAA), Native American (NA), or other ancestry (OA). Genomic and transcriptomic analyses were limited to 475 men classified as EA or AA who had data available for the analysis of interest.
(TIF)

S1 Table Self-identified race and ethnicity in TCGA prostate cancer cohort. (XLSX)

S2 Table Global genetic ancestry annotation of TCGA-PRAD patients estimated by EIGENSTRAT. (XLSX)

S3 Table Global genetic ancestry annotation of TCGA-PRAD patients estimated by STRUCTURE. (XLSX)

S4 Table Global genetic ancestry annotation of TCGA-PRAD patients estimated by LAMP. (XLSX)

S5 Table Clinical characteristics of TCGA-PRAD patients. (XLSX)

S6 Table Differential contributions of mutational signatures among AA and EA patients. (XLSX)

S7 Table Differences in mutation frequency between AA and EA patients for 21 prostate cancer-related genes. (XLSX)

S8 Table Differences in fusion event frequency between AA and EA patients. (XLSX)

S9 Table Differences in SCNA frequency between AA and EA patients. (XLSX)

S10 Table Genes differentially expressed between AA and EA patients identified by DESeq2 analysis adjusting for clinical factors. (XLSX)

S11 Table Gene sets significantly enriched in DEGs upregulated in AA tumors. (XLSX)

S12 Table Gene sets significantly enriched in DEGs downregulated in AA tumors. (XLSX)

S13 Table LncRNA genes differentially expressed between AA and EA patients identified by DESeq2 analysis adjusting for clinical factors. (XLSX)
