# Supplementary figures and images for: Integrative comparison of the genomic and transcriptomic landscape between prostate cancer patients of predominantly African or European genetic ancestry
Source: PLoS Genet. 2020 Feb 14;16(2):e1008641. doi: 10.1371/journal.pgen.1008641 (PMC7046294; doi:10.1371/journal.pgen.1008641)

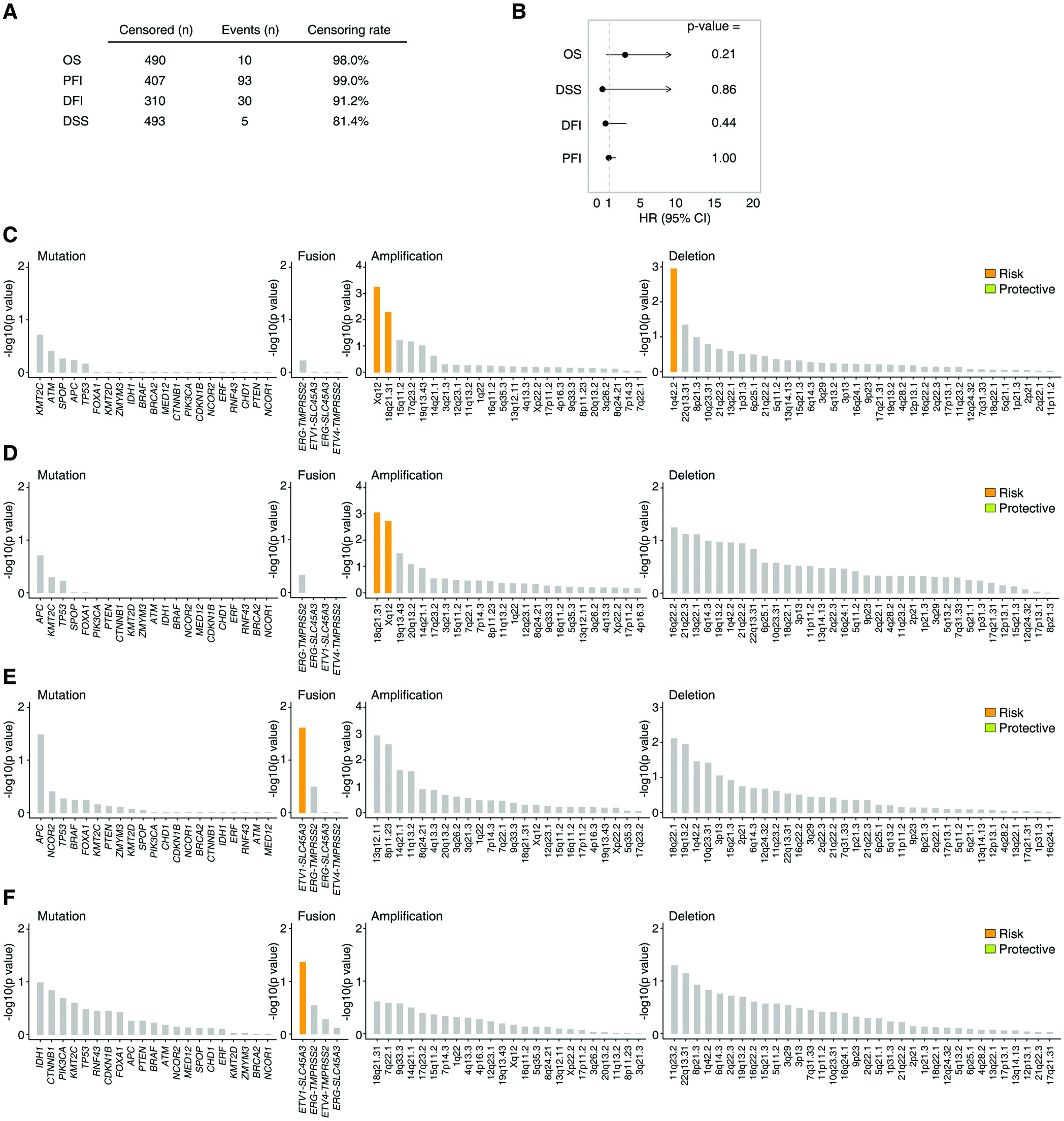

Supplement: S1 Fig — (A) Censoring rates of OS, DSS, DFI, and PFI. (B) Forest plot showing the association between African ancestry and prostate cancer survival outcomes. Dots and horizontal lines represent HR values and their 95% CIs, respectively. Arrows indicate where the CI values extend outside the range indicated. P-values were calculated from the Wald test of the coefficient for genetic ancestry in the Cox PH models. (C-F) Genomic alterations associated with (C) OS, (D) DSS, (E) DFI, and (F) PFI. The bar plots show the significance (y axis) for each genetic event of somatic mutations, transcript fusions, recurrent focal amplifications, and recurrent focal deletions. Orange and green bars represent the genetic events that had significantly higher or lower alteration frequencies among patients with worse survival outcomes, respectively. Genomic alterations with non-significant results are colored in gray. (TIF) [file pgen.1008641.s001.tif]

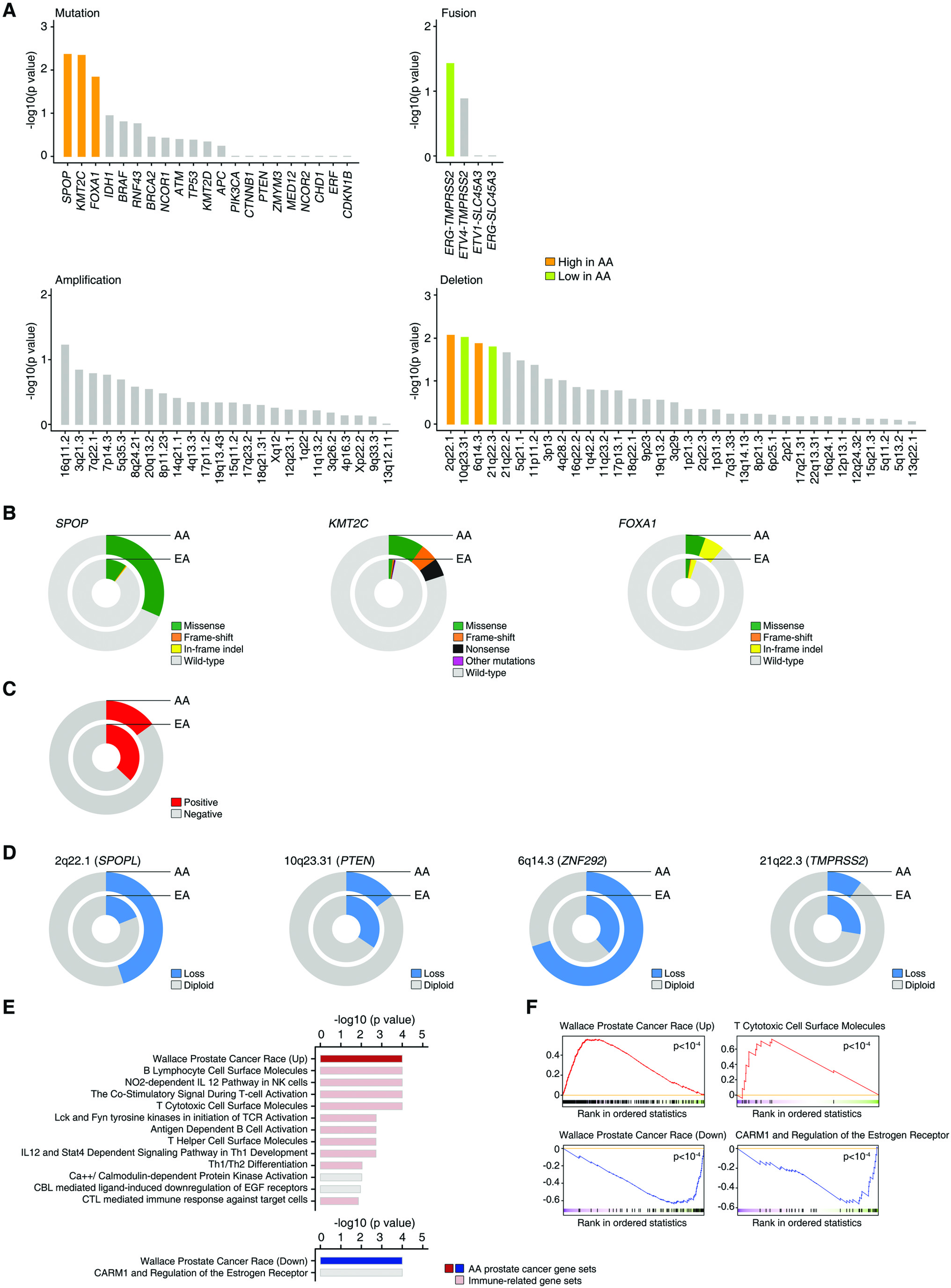

Supplement: S2 Fig — (A) Comparison of genomic alterations by genetic ancestry. The bar plots show the significance (y axis) for each genetic event of somatic mutations, transcript fusions, recurrent focal amplifications, and recurrent focal deletions. Orange and green bars represent the genetic events with alteration frequencies that were significantly higher or lower in patients of AA ancestry, respectively. Genomic alterations with non-significant results are colored in gray. (B) Mutation frequency of SPOP, KMT2C, and FOXA1 stratified by genetic ancestry. (C) Frequency of TMPRSS2-ERG fusion stratified by genetic ancestry in high risk prostate cancer patients. (D) Frequency of 2q22.1 (SPOPL), 10q23.31 (PTEN), 6q14.3 (ZNF292) and 21q22.3 (TMPRSS2) copy number alterations stratified by genetic ancestry. (E) Gene sets identified through gene set enrichment analysis that were significantly activated (upper plot; n = 13) and repressed (lower plot; n = 2) in tumors of AA men (FDR corrected p<0.1 through permutation test). Candidate gene sets were identified from the BioCarta pathway database and Wallace et al. Many gene sets pertaining to immune-related signaling were activated in AA tumors. (F) Enrichment plots of the Wallace Prostate Cancer Race (Up), T Cytotoxic Cell Surface Molecules, Wallace Prostate Cancer Race (Down), and CARM1 and Regulation of the Estrogen Receptor gene sets. (TIF) [file pgen.1008641.s002.tif]

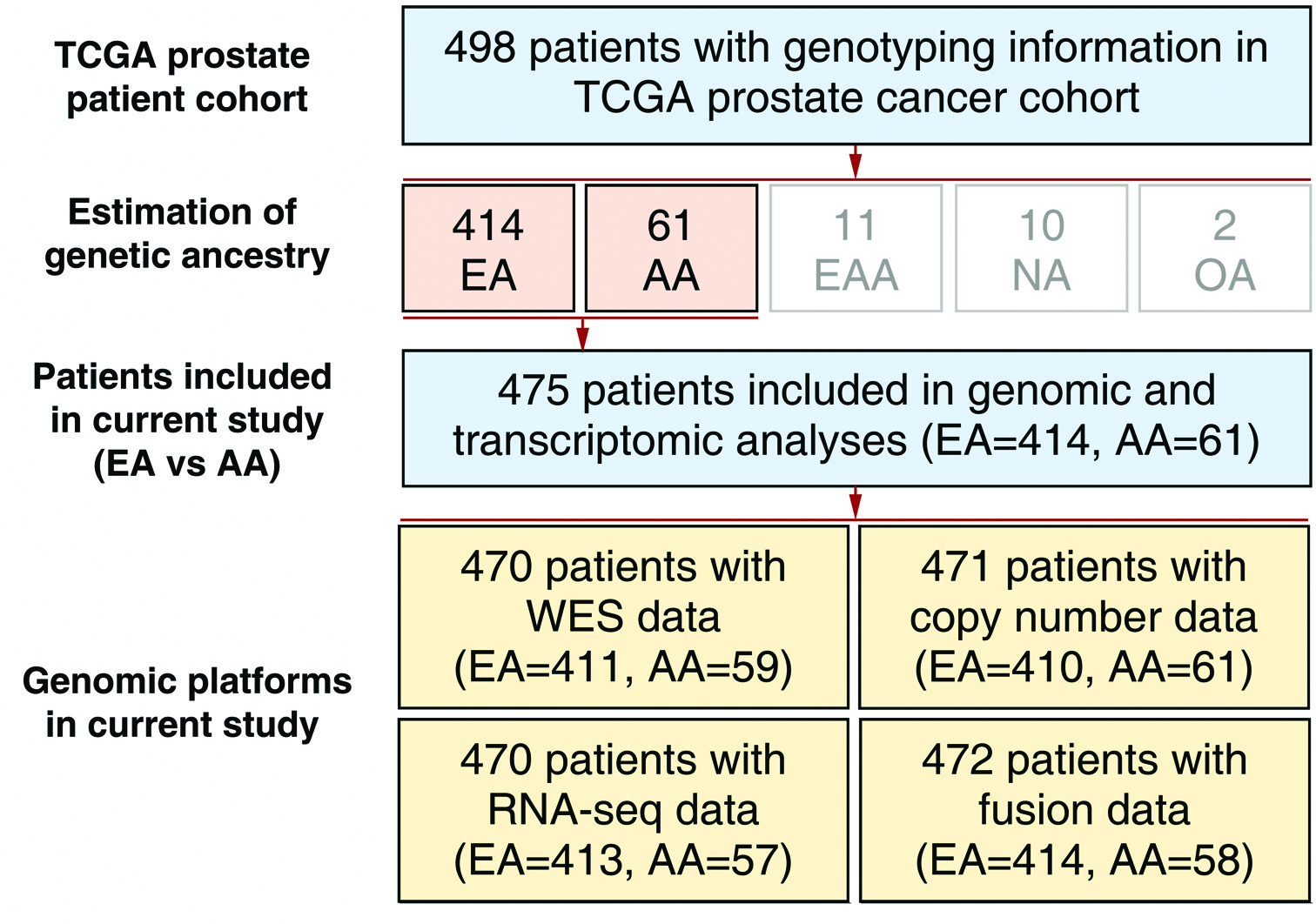

Supplement: S3 Fig — Clinical and genomic data were retrieved for 498 patients. EIGENSTRAT and a k-nearest neighbors (k-NN) classifier were applied to classify individuals as: European American (EA), African American (AA), East Asian American (EAA), Native American (NA), or other ancestry (OA). Genomic and transcriptomic analyses were limited to 475 men classified as EA or AA who had data available for the analysis of interest. (TIF) [file pgen.1008641.s003.tif]
